# Time-Domain Simulation of Along-Track Interferometric SAR for Moving Ocean Surfaces

**DOI:** 10.3390/s150613644

**Published:** 2015-06-10

**Authors:** Takero Yoshida, Chang-Kyu Rheem

**Affiliations:** 1 Department of Ocean Technology, Policy and Environment, The University of Tokyo, 5-1-5 Kashiwanoha, Kashiwa, Chiba 153-8505, Japan; 2 Institute of Industrial Science, The University of Tokyo, 4-6-1 Komaba, Meguro-ku, Tokyo 153-8505, Japan; E-Mail: rheem@iis.u-tokyo.ac.jp

**Keywords:** along-track interferometric SAR, time-domain simulation, ocean observation

## Abstract

A time-domain simulation of along-track interferometric synthetic aperture radar (AT-InSAR) has been developed to support ocean observations. The simulation is in the time domain and based on Bragg scattering to be applicable for moving ocean surfaces. The time-domain simulation is suitable for examining velocities of moving objects. The simulation obtains the time series of microwave backscattering as raw signals for movements of ocean surfaces. In terms of realizing Bragg scattering, the computational grid elements for generating the numerical ocean surface are set to be smaller than the wavelength of the Bragg resonant wave. In this paper, the simulation was conducted for a Bragg resonant wave and irregular waves with currents. As a result, the phases of the received signals from two antennas differ due to the movement of the numerical ocean surfaces. The phase differences shifted by currents were in good agreement with the theoretical values. Therefore, the adaptability of the simulation to observe velocities of ocean surfaces with AT-InSAR was confirmed.

## 1. Introduction

Along-track interferometric synthetic aperture radar (AT-InSAR) can monitor the velocities of targets. This type of radar is used for motion observations in situations such as moving target indication [1,2,3,4] or when examining ocean surface currents [5,6].

In order to support ocean observations with AT-InSAR, some simulation techniques have been developed [7,8]. The simulations are conducted for the spectra of ocean waves in SAR imagery. There have been few studies for time-domain simulations that produce raw signals for AT-InSAR in oceanic scenes taking into account movements of ocean surfaces. Therefore, we developed a simulation technique for AT-InSAR in the time domain with regard to Bragg scattering [9], which is the primary imaging mechanism for microwave backscattering on the sea surface.

The time-domain simulation technique is suitable for obtaining raw signals to examine velocities of moving targets and ocean surfaces. The signals from moving objects received by two antennas contain phase differences due to motion of the targets. The velocities of the moving objects can be estimated by the phase differences between the two focused images. Moreover, the phase differences of the focused images contains information on the radial velocity if the antenna are in the along-track interferometric configuration.

In this paper, the simulation was developed by improving the time-domain simulation technique of Yoshida *et al.* [10]. The simulation obtains time series of raw signals for SAR images to take into account ocean surfaces moving in time. Another feature of the simulation is that the computational grids of the simulation are smaller than the wavelength of the Bragg resonant waves in order to adequately calculate microwave backscattering. By applying the simulation to two antennas, we designed the AT-InSAR simulation to acquire raw signals in the time domain and to reproduce Bragg scattering.

To validate the simulation, compressed azimuthal signals as SAR data were simulated for the Bragg resonant wave and irregular waves with currents. The phase differences were compared with the theoretical values to show the accuracy of the simulation. The present work addressed fundamental aspects of simulation for observing moving ocean surfaces with AT-InSAR. In the future, it is expected that the simulation will be used to support AT-InSAR observations such as tidal currents and ocean surface velocities.

## 2. Simulation Method

### 2.1. AT-InSAR Signal

The numerical simulation developed by Yoshida *et al.* [10] mainly considers effects of motion-induced modulation as well as Bragg scattering. It is designed in the time domain so that the simulated raw signals have modulations due to moving ocean surfaces. In addition, the simulation is based on Bragg scattering because the computational grids are smaller than Bragg resonant waves.

**Figure 1:**
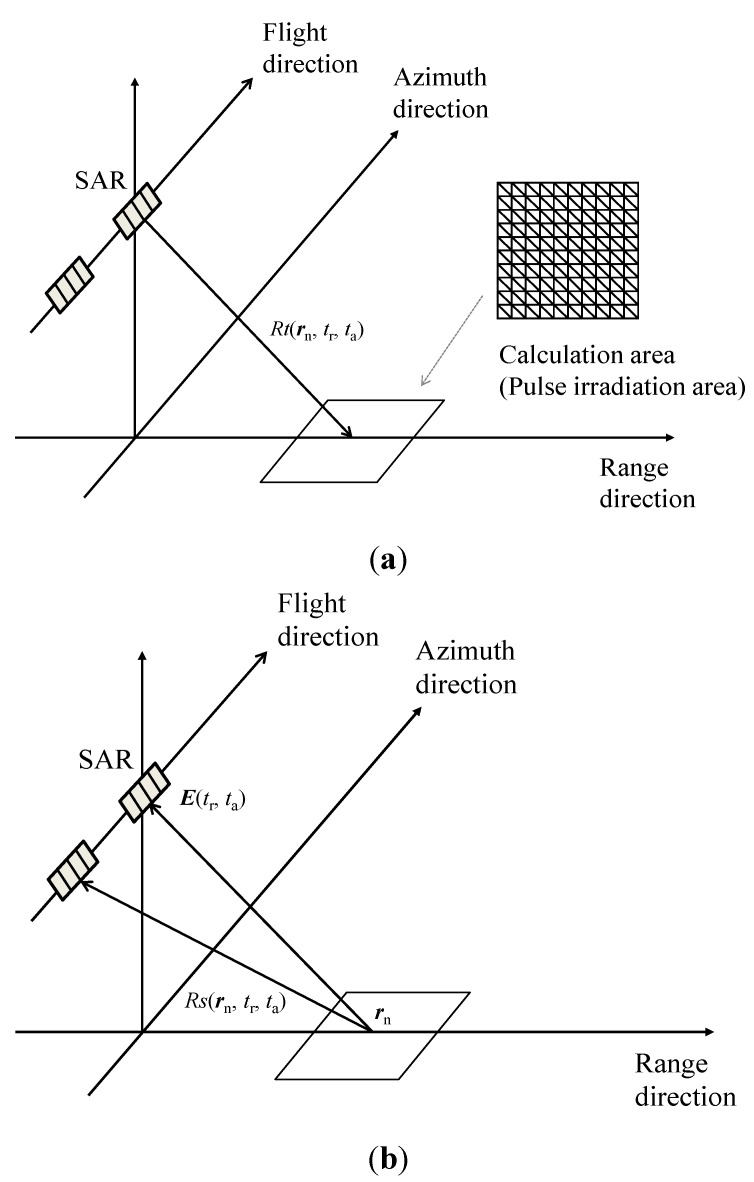
Schematic images of the AT-InSAR simulation. (**a**) transmitted microwave; (**b**) received microwave.

In this paper, the simulation technique has been revised for AT-InSAR simulation with two antennas. As shown in Figure 1a, the pulse irradiation area is the calculation area, which is divided into a computational grid composed of triangle elements. The calculation of microwave backscattering is based on physical optics (PO) [11]. PO is computational efficient because each computational grid element is considered independently. It enables large-scale simulations with small computational grids.

**Figure 2:**
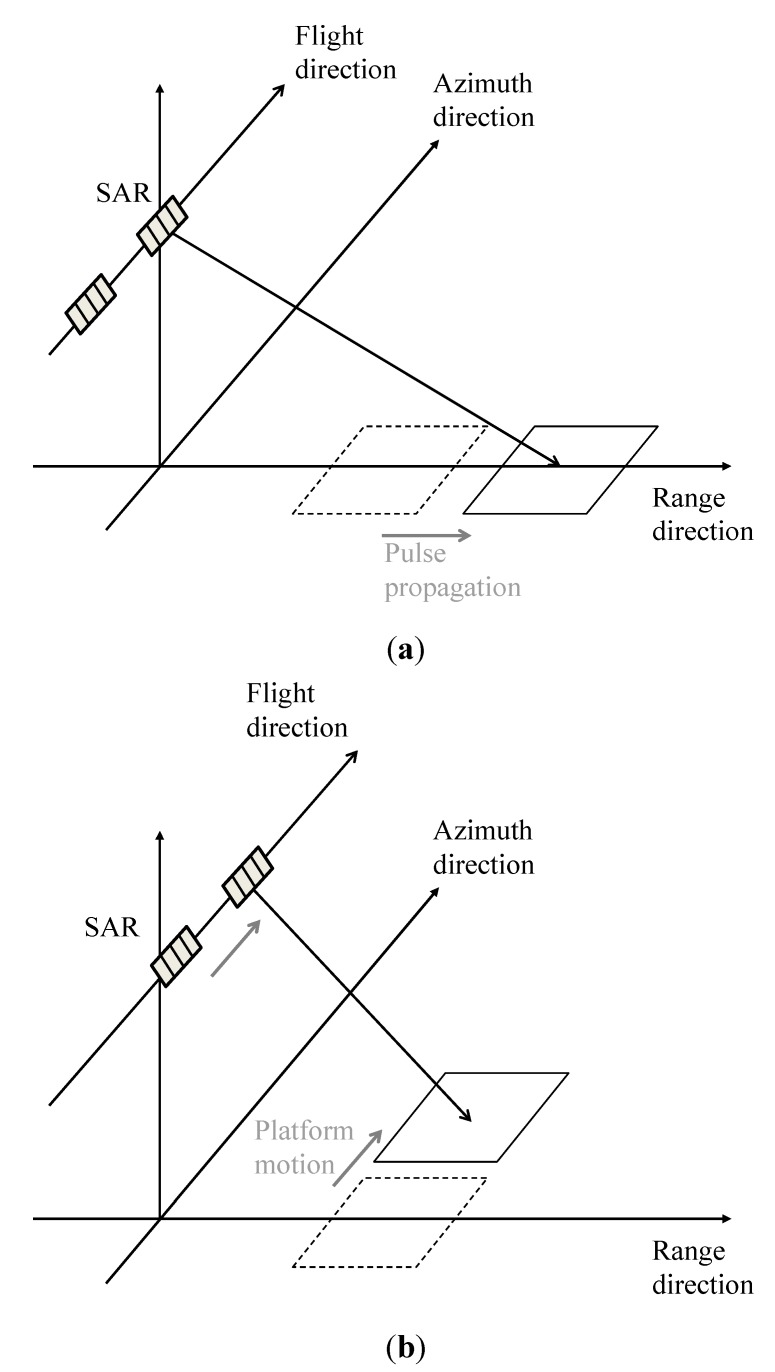
Schematic images of altering the locations of the calculation areas. (**a**) pulse propagation in the range direction; (**b**) platform motion in the azimuth direction.

In this simulation, AT-InSAR is in “standard” mode [12]; that is, the master antenna transmits microwaves, then the master and slave antennas receive backscattered microwaves, as shown in Figure 1a,b. Figure 2a,b shows that the location of the calculation area changes with pulse propagation and platform movement. The time series of microwave backscattering are obtained as raw signals of AT-InSAR by altering the locations of the calculation areas at each sampling time of the range and azimuth directions.

The simulation method for calculating microwave backscattering is as follows: at first, let us consider the case of a CW (continuous wave) radar. Based on PO, the scattering electric field **E** is expressed as the following discrete equation:

(1) $$E=-i2\pi f\mu \sum_{n=1}^{N}J\frac{\exp(i2\pi fR_{n}/c)}{4\pi R_{n}}A_{n}$$

where *R_n_* is the distance between the antenna and each computational grid element. *f* is the frequency of the transmitted microwave. *c* is the light velocity. *i* is the imaginary unit. μ is the magnetic permeability. *N* is the number of computational grids. The area of the computational grid is *A_n_*.

When observation targets are treated as perfect conductors (e.g., ocean surface), the surface electric current **J** is given by:

(2) J=2n×H

where **n** is the surface normal vector of each computational grid element, **H** is the incident magnetic field that is:

(3) $$H=A\exp\left(i2\pi fR_{n}/c\right)$$

where **A** is the amplitude that is composed of (range, azimuth, altitude) directions.

From Equation (1) with Equations (2) and (3), we can obtain that:

(4) $$E=-i2\pi f\mu \sum_{n=1}^{N}\frac{2n\times A\exp(i2\pi f\cdot 2R_{n}/c)}{4\pi R_{n}}A_{n}$$

The exponential term expresses the phase of backscattered signals. Next, in the case of chirp pulse for SAR, the magnetic incident magnetic field is:

(5) $$H=A\exp\left(i2\pi f_{c}\frac{R_{n}}{c}\right)\cdot \exp\left(i\alpha {\left(\tau -\frac{R_{n}}{c}\right)}^{2}\right)$$

where *f_c_* is the center frequency of the transmitted chirp pulse and α is the linear chirp rate. *τ* is given by the following equation:

(6) $$\tau =R_{c}/c$$

where *R*_c_ is the distance from the antenna and the center of the transmitted pulse on the ground. In order to apply the simulation for AT-InSAR, scattering electric fields are treated as functions **r***_n_*, *t*_r_, and *t*_a_, where **r***_n_* is the position of the nth grid element, *t*_r_ and *t*_a_ are the times sampled in the range and azimuth directions, respectively. Now we introduce *R_t_* and *R_s_* that are the distances between transmit and received antennas and each computational grid element. Then combining Equations (4)–(6), the scattering electric field of AT-InSAR is expressed as follows:

(7) $$\begin{array}{l} E(r_{\text{n}},t_{\text{r}},t_{\text{a}})=-i2\pi f_{c}\mu \sum_{n=1}^{N}2n\times \sqrt{\frac{P}{\eta }}\exp\left(i2\pi f_{c}\frac{R_{t}(r_{\text{n}},t_{\text{r}},t_{\text{a}})+R_{s}(r_{\text{n}},t_{\text{r}},t_{\text{a}})}{c}\right)\\ \exp\left(i\alpha {\left(\frac{2R_{c}(t_{\text{r}},t_{\text{a}})}{c}-\frac{R_{t}(r_{\text{n}},t_{\text{r}},t_{\text{a}})+R_{s}(r_{\text{n}},t_{\text{r}},t_{\text{a}})}{c}\right)}^{2}\right)\frac{A_{n}}{4\pi R_{s}(r_{\text{n}},t_{\text{r}},t_{\text{a}})} \end{array}$$

where η = (μ/ε)^0.5^, ε is the permittivity. For the signals of master antenna, *R*_s_ is equal to *R_t_*. Considering the power density **P** including the antenna beam pattern and the spreading loss of the transmitted antenna, it is given by the following equation. Here, it is defined as a function of *β*_H_ and *β*_V_, which are the angles between the incident angles and each computational grid element for simplicity:

(8) $$P=\frac{P_{0}}{4\pi {R_{t}}^{2}}{\left|\text{sinc}\left(\frac{\pi D_{H}}{\lambda_{e}}\sin(\beta_{H})\right)\right|}^{2}{\left|\text{sinc}\left(\frac{\pi D_{V}}{\lambda_{e}}\sin(\beta_{V})\right)\right|}^{2}$$

where **P**_0_ is the magnitude of the power density. *D* is the length of the antenna. The subscripts H and V show that horizontal and vertical planes. λ*_e_* is the wavelength of microwave.

From Equation (7), the time series of the received signals are obtained as the summations of the scattering electric fields of all computational grid elements. The calculation area moves with sampling time for range and azimuth directions. Then, the time series of SAR signals are obtained. Finally, SAR images with fine resolutions are obtained by applying range and azimuth compression using FFT (Fast Fourier Transform). As compression techniques, the convolutions are performed in the frequency domain between the raw data and the reference signal, which is a complex conjugation signal from a point target. The phase differences are calculated by the received signals of master and slave antennas. The details are explained in the next section.

### 2.2. Interferometry

**Figure 3:**
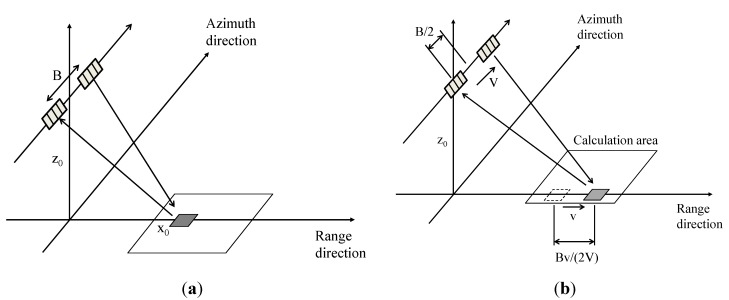
Relationships between AT-InSAR and a moving target. (**a**) Before target movement; (**b**) After target movement.

The phase differences caused by ocean surfaces are described theoretically in the following equations. An approximate equation assumes incident angles are considered as the same before and after the movements of the scattering object in the case of high altitude of the antennas. Ocean surfaces are applied to the equation because they are assumed as scattering objects that are uniform random surfaces. The line-of-sight velocity of the target and the phase difference are:

(9) $$\delta \phi =\frac{4\pi }{\lambda }\frac{B}{2V}v\sin\theta$$

where δφ is the phase difference between the master and slave antennas. λ is the wavelength of microwave. As shown in Figure 3, *B* is the base line, *V* is the platform velocity, and *v* is the velocity of the target. θ is the incident angle.

When *E*_1_ and *E*_2_ are the complex scattering electric fields as signals of the master and slave antennas, the phase difference between them is described in Equation (10):

(10) $$\delta \phi =arg(E_{1}{E_{2}}^{*})$$

here, *arg* is the argument function with range from −π to π. The asterisk in Equation (10) denotes the conjugate of the complex signal.

### 2.3. Numerical Sea Surface

Numerical sea surfaces of irregular waves are generated by wave spectra. It is expressed as summations of linear waves with random phase *a_ij_*. The elevation of irregular sea surfaces is given by:

(11) $$z\left(x,y,t\right)=\sum_{j=1}^{N_{2}}\sum_{i=1}^{N_{1}}\cos\left(k_{i}x\cos\Phi_{j}+k_{i}y\text{sin}\Phi_{j}-\omega_{i}t+a_{ij}\right)\sqrt{2S(\omega_{i})\Delta \omega_{i}}$$

where the number of component waves is *N*_1_, the number of directions of component waves is *N*_2_. *x* and *y* are the distance variables, *t* is the time variable. ω_i_, *k*_i_ are the angular frequency and the wave number of component waves, respectively. *Δ*ω is the frequency intervals. Φ is the angle between the wave directions and the *x*-direction. Here, *x* and *y* are range and azimuth directions in the simulation.

For deep water waves, there is the dispersion relationship expressed as following equation:

(12) $$\omega =\sqrt{gk}$$

Pierson and Moskowitz spectrum [13] is used to reproduce a high-frequency wave. The wave spectrum *S* is

(13) $$S\left(\omega \right)=\left(8.1\times 10^{-3}\right)g^{2}\omega^{-5}\exp\left(-0.74{\left(\frac{g}{\omega U}\right)}^{4}\right)$$

where ω is the angular frequency of the spectra. *g* is the gravitational constant. *U* is the wind speed at 10 m height on the ocean surface.

## 3. Results

In order to apply the simulation to ocean surface observations, simulations of SAR signals in the azimuth direction were carried out for numerical ocean waves with ocean surface current. Section 3.1 describes the case of a single wave that is considered as a Bragg resonant wave. Then, irregular waves are simulated in Section 3.2. In order to validate the simulation, phase shifts caused by the addition of current were examined.

### 3.1. Bragg Resonant Wave

It is known that Bragg scattering governs microwave scattering. Accordingly, Bragg resonant waves were numerically generated as ocean surface waves. Here, the incident angle is 40 degrees, and the radar wavelength is 0.235 m; thus, the calculated wavelength of the Bragg resonant wave is 0.186 m. The amplitude is set as 0.002 m. The wave direction is toward the radar. It means that the wave propagates along the range direction.

The simulation conditions are listed in Table 1. The simulations were performed for wave and wave with current. If the uniform current *U*_c_ is added linearly, the angular frequency in Equation (11) expressed as ω *− kU*_c_*.* Here, *U*_c_ is set as 0.5875 m/s and its direction is parallel to the range direction. Note that the simulation was only conducted for SAR signals in the azimuth direction. The azimuth signals were remarked to show phase differences of waves traveling to the range direction so that the calculation area is assumed as small in the range direction. The length of the range calculation area is the same as range resolution.

**Figure 4:**
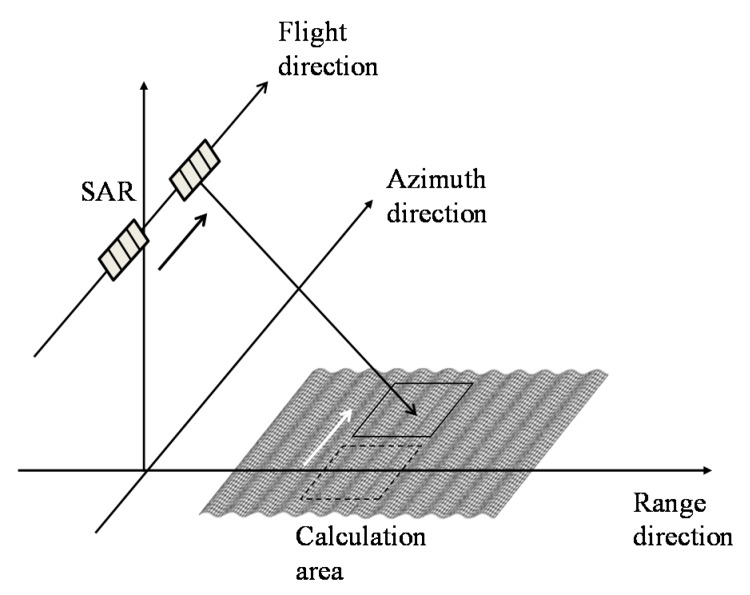
Schematic illustrations of azimuth signal simulations for numerical wave cases.

**Figure 5:**
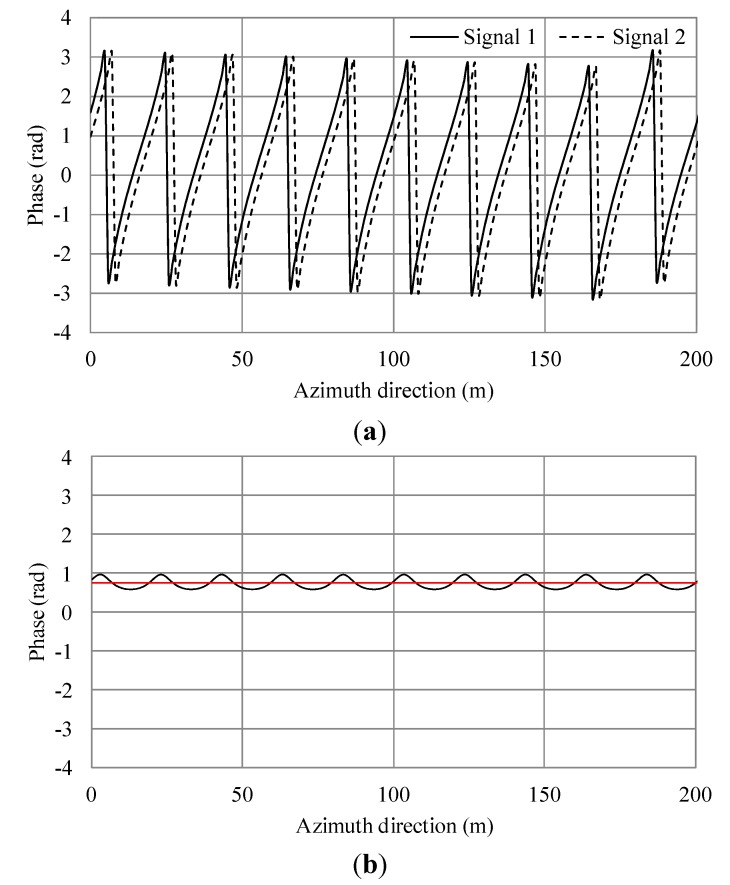
Simulation results of a single Bragg resonant wave. (**a**) Phases of simulated SAR signals of master and slave antennas in the azimuth direction; (**b**) Phase difference of (a). The black line shows the phase differences. The red line shows the average.

**Figure 6:**
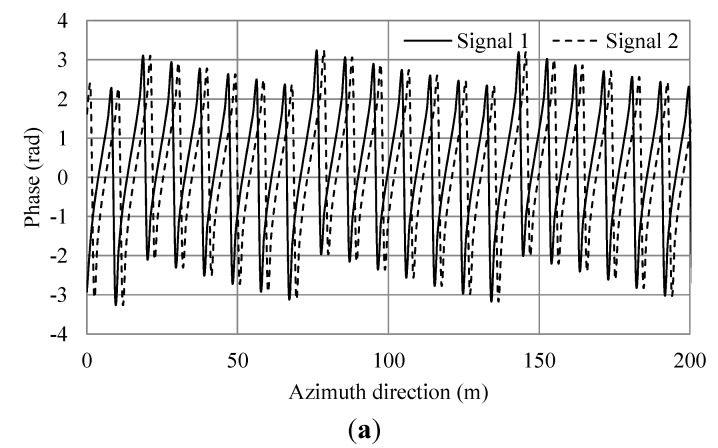

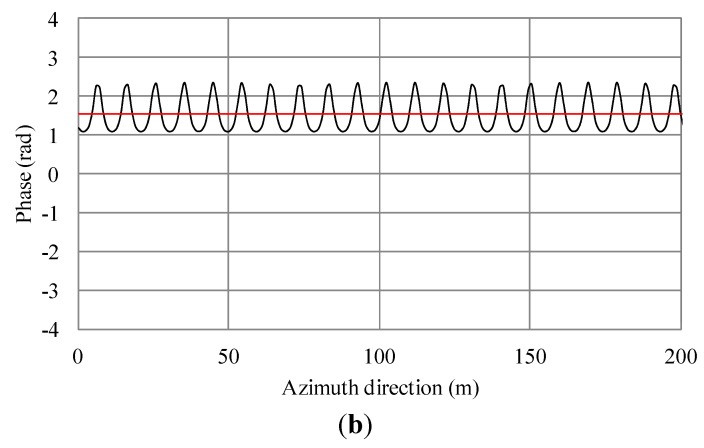
Simulation results of a single Bragg resonant wave with an additional current. (**a**) Phases of simulated SAR signals of master and slave antennas in the azimuth direction; (**b**) Phase difference of (a). The black line shows the phase differences. The red line shows the average.

Figure 4 depicts the schematic illustration of simulation for Bragg resonant waves. The Bragg resonance enhances backscattered signals due to correspondence of their phases. Figure 5 and Figure 6 show the SAR signals of the phases from two antennas and their phase differences. Figure 5a,b is the results for a single wave, on the other hand Figure 6a,b shows the results for addition of a current to a wave. As shown in Figure 5a, the phases of the SAR signals vary with the sampling time of the azimuth signals because the Bragg resonant wave moves while the antenna moves in the azimuth direction to obtain the SAR signals.

**Table 1:** Simulation conditions.

Parameter	Value
Radar frequency	1.275 GHz
Radar wavelength	0.235 m
Polarization	HH
Incident angle	40 deg
Range resolution	4.5 m
Azimuth resolution	3 m
Pulse duration	0.2 μs
Pulse reputation frequency	50 Hz
Chirp rate	250 × 10^12^ Hz/s
Chirp band width	50 MHz
Sampling frequency	255.3 MHz
Altitude of platform	1500 m
Velocity of platform	58.75 m/s
Calculation area (small range case)	Range: 100 (4.7)m Azimuth: 80 m
Scale of computational grid	0.047 m
Range beam width	10.0 degree
Range antenna length	1.2 m
Azimuth beam width	2.0 degree
Azimuth antenna length	6.0 m
Base line	4.7 m

The phase differences of the ocean surface are due to ocean surface currents, wind surface velocity, phase velocity of the Bragg resonant wave and the orbital motion of ocean waves [14,15]. As shown in Figure 5a, the phase differences are linked to phase velocity of the Bragg resonant wave. The phase difference also depends on the distance between the antennas and the sea surface. Currents can be estimated relatively by comparing to the phase with no current. Figure 6b shows the phase shift due to the current. Comparing Figure 5b with Figure 6b, the average values are 0.738 and 1.531 radians, respectively. The difference is 0.793. Here, the theoretical phase shift calculated using Equation (9) is 0.8. Therefore, the simulation result agrees well with the theoretical value with the error within 1%.

### 3.2. Irregular Wave with Current

We simulated irregular waves with currents traveling in the range direction in order to show the application to irregular waves with currents. The simulation conditions were the same as Section 3.1 except for the numerical surfaces. Irregular waves were produced numerically based on Section 2.3. The details of the numerical wind waves were as follows. The range of wavelengths was 0.3 m to 20 m. The number of spectral divisions was 50. Wave direction had five components: 0°, ±10°, and ±20°, where 0° is toward to the antenna. In this section, three wind speeds *U* = 5, 7.5, 15 m/s in Equation (13) were investigated for the cases with and without current. Here, the current *U*_c_ was 0.5875 m/s, which is the same as Section 3.1.

**Figure 7:**
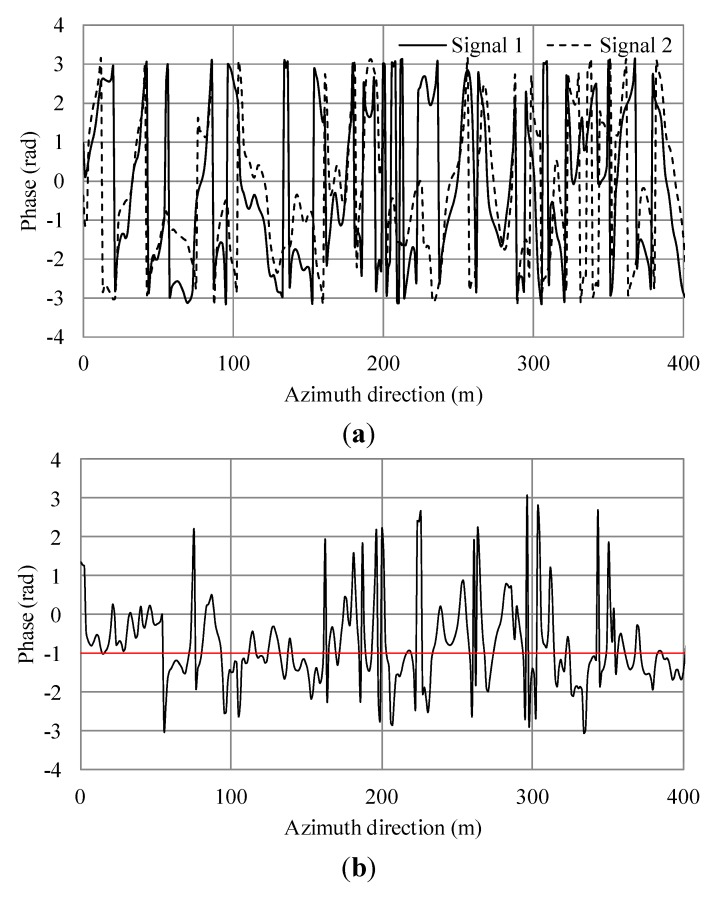
Simulation results of irregular waves for wind speed 5 m/s. (**a**) Phase of simulated SAR signals of master and slave antennas in the azimuth direction; (**b**) Phase differences of (a). The black line shows the phase differences. The red line shows the average.

**Figure 8:**
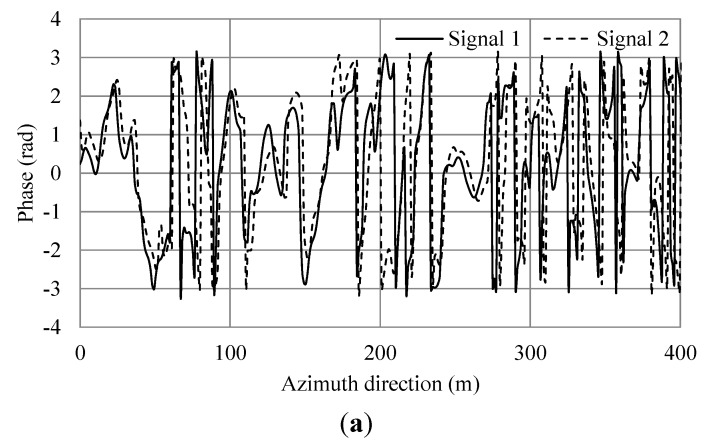

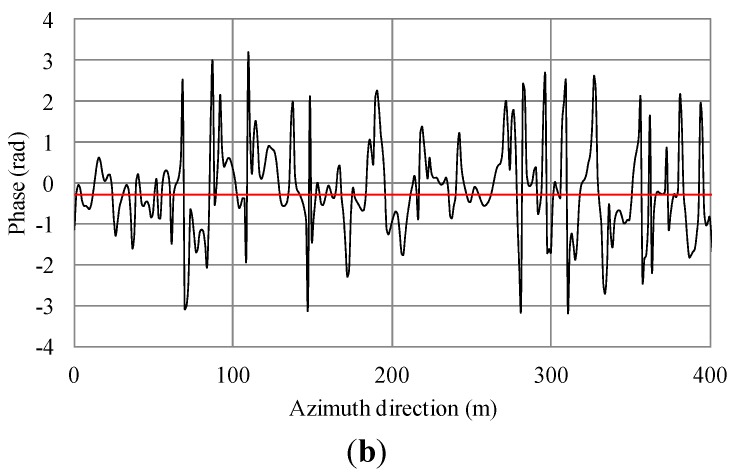
Simulation results of additional current and irregular waves for wind speed 5 m/s. (**a**) Phase of simulated SAR signals of master and slave antennas in the azimuth direction. (**b**) Phase differences of (a). The black line shows the phase differences. The red line shows the average.

**Figure 9:**
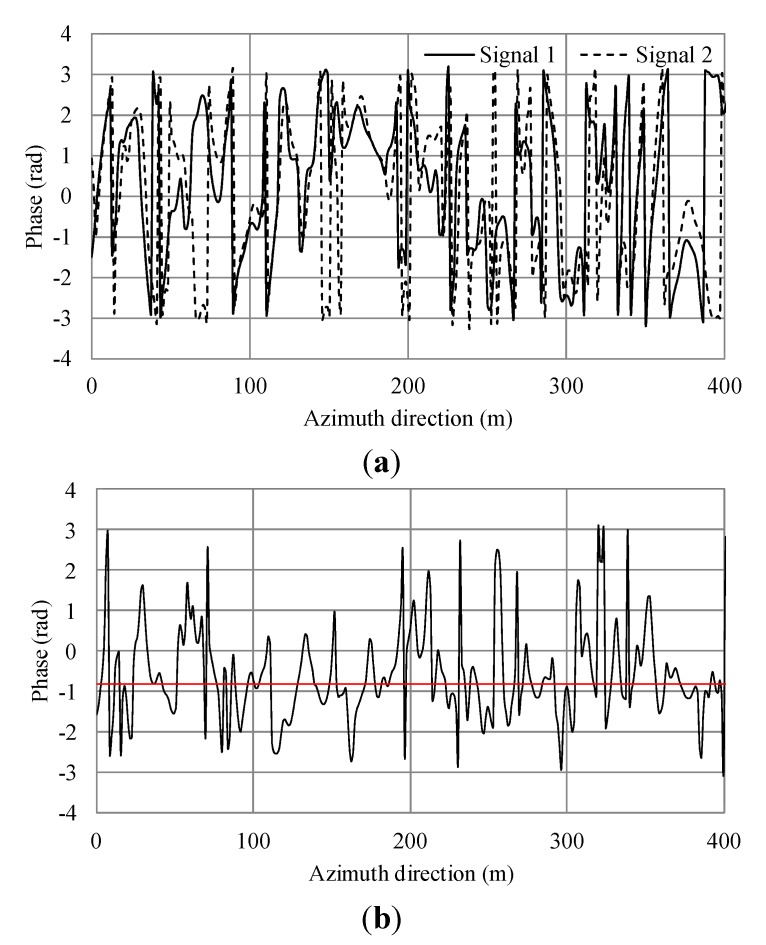
Simulation results of irregular waves for wind speed 7.5 m/s. (**a**) Phase of simulated SAR signals of master and slave antennas in the azimuth direction; (**b**) Phase differences of (a). The black line shows the phase differences. The red line shows the average.

**Figure 10:**
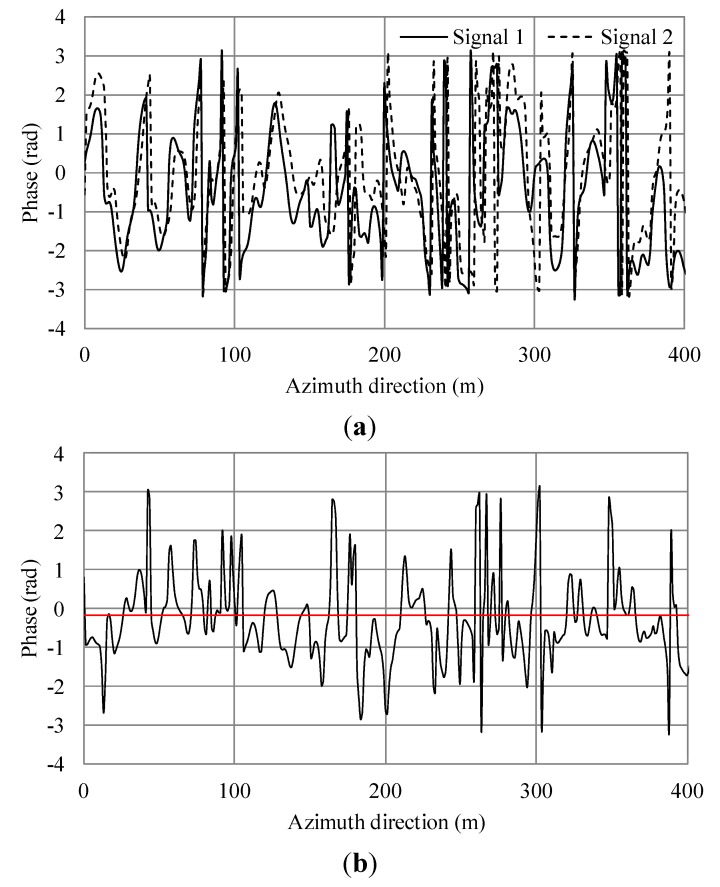
Simulation results of additional current and irregular waves for wind speed 7.5 m/s. (**a**) Phase of simulated SAR signals of master and slave antennas in the azimuth direction. (**b**) Phase differences of (a). The black line shows the phase differences. The red line shows the average.

**Figure 11:**
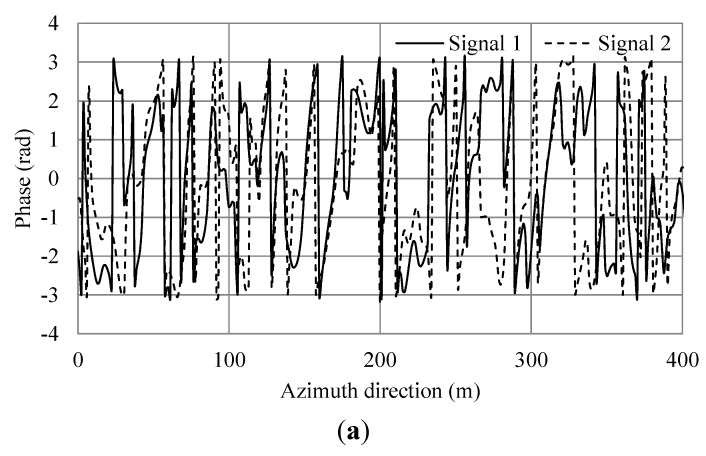

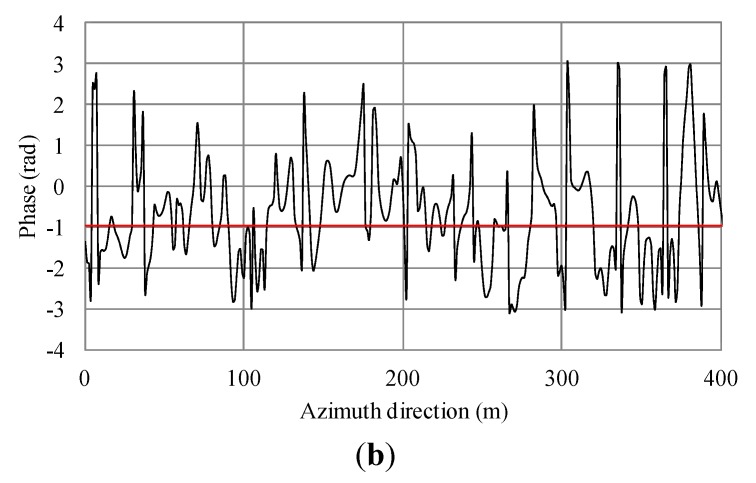
Simulation results of irregular waves for wind speed 15 m/s. (**a**) Phase of simulated SAR signals of master and slave antennas in the azimuth direction. (**b**) Phase differences of (a). The black line shows the phase differences. The red line shows the average.

**Figure 12:**
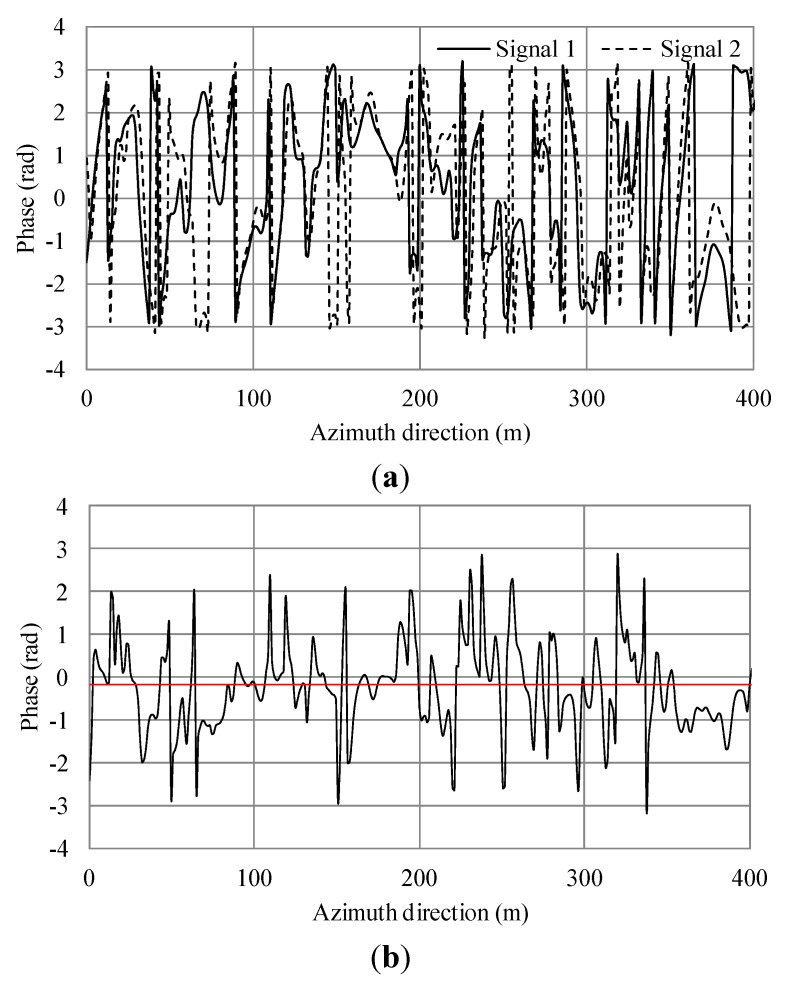
Simulation results of additional current and irregular waves for wind speed 15 m/s. (**a**) Phase of simulated SAR signals of master and slave antennas in the azimuth direction. (**b**) Phase differences of (a). The black line shows the phase differences. The red line shows the average.

The results of the simulated SAR signals in the azimuth direction for three wind speeds are shown in Figure 7a, Figure 8a, Figure 9a, Figure 10a, Figure 11a and Figure 12a, that are SAR signals of master and slave antennas. The phase differences between two antennas are displayed in Figure 7b, Figure 8b, Figure 9b, Figure 10b, Figure 11b and Figure 12b. The results represent the phase differences for uniform surface current, phase velocity of the Bragg resonant wave, and orbital motions of irregular waves. The wind surface velocity is neglected in the numerical surfaces of this simulation. The fluctuations seen in these figures are due to irregular backscattering microwave effects, in other words, microwave backscattering randomly occurs on irregular wave surfaces. It can be treated as speckle noise.

Here, in order to analyze the average value of cyclic phases, we first calculated the median values. Then unwrapping was conducted for the range from median + π to median − π. Finally, the average value within this range was calculated. This method reduces the influence of cyclic phases. If we obtain an average to evaluate phase differences that includes speckle noise, the average value is affected by cyclic phases. Therefore, we adopted this method to calculate average values of phase shifts caused by currents.

From Equation (9), the theoretical phase shift in this condition is 0.8. For the case of wind speed of 5 m/s, the averages of Figure 7b and Figure 8b were −1.015 and −0.281 radians, respectively. The difference of between them was 0.734 and the error 8.25% compared to the theoretical value. For a wind speed of 7.5 m/s, the averages of Figure 9b and Figure 10b were −0.819 and −0.173 radians, respectively. The difference of between them was 0.646 and the error 19.25%. For a wind speed of 15 m/s, the averages of Figure 11b and Figure 12b were −0.963 and −0.166 radians, respectively. The difference between them was 0.797 and the error 0.375%. The phases were shifted by the currents and there were errors due to the orbital motions of irregular waves that were strongly dependent on the situation of the ocean waves. Nevertheless, these errors are acceptable because actual ocean observation by radar also contains some errors due to noise. The simulation results show that the phase shifts caused by the currents were in good agreement with the theoretical values. Thus, the simulation was validated and it was confirmed that the simulation was adaptable for simulations of AT-InSAR in ocean areas. Robustness for application to various sea states was also shown. However, the model used in this research is ideal, e.g., realistic radar system noise is not considered. This model also assumes a straight flight path. One of the advantages of a time-domain simulation is that it easily offers the opportunity to simulate arbitrary platform motion. The results of this study demonstrated the fundamental simulation technique and we plan to address these issues in future work.

## 4. Conclusions

We have developed an AT-InSAR time domain simulation for application to observations of moving ocean surfaces. The time-domain simulation can acquire raw signals with regard to moving objects. In addition, the simulation was improved by being a simulator based on Bragg scattering. Therefore, this simulation is suitable for AT-InSAR observations of moving ocean scenes.

The simulations were conducted for a Bragg resonant wave and irregular waves taking into account the effect of ocean currents. For a Bragg resonant wave, the phase difference between two antennas shows the phase velocity of the Bragg resonant wave. Moreover, the phase shift due to the added current agrees well with the theoretical value. Irregular waves and currents are also simulated to show phase shifts caused by currents. Compared to the results with and without current, the phases of the irregular waves with current were shifted by the current. The phase shifts due to the currents had several errors compared to the theoretical value. However, the results were acceptable for validation of the simulation because noise is also seen in actual measurement data due to orbital motions of ocean waves. From the results, we confirmed that the simulation is adaptable for observations of ocean surfaces. The simulation will be used to evaluate the ocean observations by AT-InSAR.
